# Morphology and Anti-Corrosive Performance of Cr(III) Passivated Zn–Fe Alloy Coating on NdFeB Substrate

**DOI:** 10.3390/ma15217523

**Published:** 2022-10-27

**Authors:** Ba Li, Xiaoshun Zhou, Xiaoping Chen, Song Fu, Xiangdong Wang, Dongliang Zhao

**Affiliations:** 1 College of Chemistry and Life Sciences, Zhejiang Normal University, Jinhua 321004, China; 2 Central Iron and Steel Research Institute Co., Ltd., Beijing 100081, China; 3 Zhejiang Innuovo Magnetics Co., Ltd., Dongyang 322118, China

**Keywords:** Nd-Fe-B, pure Zn coatings, Zn–Fe alloy coatings, trivalent chromium passivation, corrosion resistance

## Abstract

In this study, low-iron Zn–Fe alloy coatings and pure Zn coatings, with or without trivalent chromium passivation treatment, were electrodeposited onto a sintered NdFeB magnet from a weak acid chloride bath. The surface morphology and structure of the coatings were then examined using the X-ray diffraction, a scanning electron microscope and 3D white-light interfering surface analysis. Meanwhile, the electrodeposition behavior and anti-corrosive properties of the coatings were investigated using cyclic voltammetry, potentiodynamic polarization, electrochemical impedance spectroscopy, and natural salt spray tests. The results indicate that a passivated Zn–Fe alloy coating with a 0.9 wt.% Fe content provided much better corrosion resistance than a pure Zn coating and could provide both anodic protection and physical barrier function in the NdFeB substrates. The Fe element in Zn–Fe alloy coating was predominantly in solid solution in η-phase and small amounts in elemental form, which was beneficial to acquire a compact coating and passivation film. Finally, the passivated Zn–Fe alloy coating withstood 210 h against a neutral 3.5 wt.% NaCl salt spray without any white rust, which was 3–4 times longer than the pure Zn coating.

## 1. Introduction

Sintered NdFeB magnets have been widely used in industry applications, such as electronics, communications and automation, due to their excellent magnetic properties [1,2]. However, magnets present poor corrosion resistance in various environments due to the high reactivity of Nd and the existence of a multiple-phase structure, which strictly limits their further applications [3,4,5,6]. According to our knowledge, many attempts have been made to improve the corrosion resistance of NdFeB magnets, such as alloy addition and surface treatment [7,8,9]. Compared with alloy addition, surface treatments, such as monometal [10,11,12,13,14], nonmetal [15,16], alloy [17,18,19,20,21] or composite [22,23,24,25,26,27] coatings, can effectively improve the corrosion resistance of NdFeB magnets without reducing their magnetic fields. However, in all of these coatings and their preparation methods, electrodeposited metal coatings are still commonly applied in industry to this day. Compared to other surface treatments, they are suitable for large-scale production due to their lower cost and easier control. Among them, electroplating zinc coatings account for a large part of the industry [10,11].

As NdFeB products are used in a growing number of fields, the requirements for coatings are continuously enhanced. Therefore, the corrosion resistance of conventional zinc coatings is not satisfactory in severe atmospheric conditions. Meanwhile, the excellent properties of coatings by Zn-iron group alloys, particularly Zn–Fe alloys, make them very promising materials as alternatives for zinc coatings of steel products [28,29]. Zn–Fe alloy coatings have many excellent properties, such as excellent corrosion resistance, prominent paintability and good welding properties in comparison to the zinc coating [30,31].

According to the type of electroplating solutions, Zn–Fe alloy coatings can be divided into alkaline zincate, acid sulfate and chloride systems [32,33,34,35]. An alkaline zincate system is difficult to directly plate onto the surface of the porous NdFeB substrate due to its low current efficiency (about 60%), which causes severe cathodic hydrogen evolution, reducing adhesion strength. Meanwhile, the sulfate system is also not appropriate for the magnet because of its high iron content in the coating [36]. Although Long et al. acquired a black chromate (Cr (VI)) conversion film on the Zn–Fe coating (0.58 wt.% Fe) with an acid sulfate bath, the pH of their plating solution (pH = 2.5) is too low to use on NdFeB [37]. Moreover, Cr (VI) chemicals are classified as carcinogens, and the professional standard has prohibited their usage [38], while the trivalent chromium (Cr (III)) passivation treatments are considered to be commercially acceptable alternatives to the conventional Cr (VI) treatments, and a number of attempts have been implemented on the zinc coatings [39,40,41,42,43].

The chloride system can do all kinds of conventional passivation, and the bath has a high current efficiency (>95%), which is believed to fit NdFeB electroplating [36]. However, there is little research on the preparation of Zn–Fe alloy coatings with Cr (III) passivation on NdFeB magnets. Structural and phase composition are the main factors that determine the properties of the coating and the passivation film on the specimen, depending on the composition of the electrodeposition and the passivation solution. Therefore, Zn–Fe alloy coatings with Cr (III) passivation on sintered NdFeB substrates are worthy of detailed research, especially from a light acid chloride solution. In this study, the surface morphology and corrosion resistance of the electrodeposited zinc and Zn–Fe alloy coatings on NdFeB-based substrates, with or without Cr (III) passivation treatment were studied. Then, the anticorrosion mechanism of the coatings was analyzed and discussed.

## 2. Experimental Section

### 2.1. Materials

The sintered NdFeB specimens used for this investigation (N52H, Zhejiang Innuovo Magnetics Co., Ltd., Dongyang, China, in a demagnetized state during electroplating) were in disk form with diameter and thickness of 12.0 mm and 2.0 mm, respectively. The coercivity of the commercial magnet is 1018 KA/m, and the maximum magnetic energy product is 422 KJ/m^3^. A zinc plate of 99.99% purity was used as an anode on the side of the electrolytic cell. In addition, a new specimen was used in each experiment.

### 2.2. Pretreatment of the Magnet

First, the NdFeB substrate was sequentially polished with a waterproof abrasive paper from a grit of 400# to 1200#. After washing by the deionized water, the specimen was degreased in a mixed solution containing 10 g/L metal powders and 40 mL/L alcohol at 50 °C for 2–3 min, and then cleaned with hot and cold deionized water, respectively. In order to remove the oxide film from the magnet surface, the pretreated specimens described above were dipped into a HNO_3_ solution with a volume fraction of 3.0% for 3–5 s at room temperature. Before electroplating, the NdFeB magnets were activated at room temperature by the H_2_SO_4_ solution with a volume fraction of 0.5% for 4–6 s.

### 2.3. Electrodeposition Process

The optimized bath composition and electrodeposition parameters for both pure Zn and Zn–Fe alloys are given in Table 1. The experimental electrolyte was prepared from analytical grade material. In particular, the additive consisted of the main, carrier and accessory brighteners. Moreover, the main purpose of adding the C_6_H_11_NaO_7_, C_4_O_6_H_4_KNa and ascorbic acid was to stabilize the Fe^2+^ ions in the bath. Complexing agents (C_6_H_11_NaO_7_ and C_4_O_6_H_4_KNa) were used to delay the conversion of Fe^2+^ to Fe^3+^, while the reducing agent (ascorbic acid) was used to reduce the oxidized Fe^3+^ to Fe^2+^. Using the electroplating bath, as shown in Table 1, we can easily obtain the Hull cell plates that were entirely reflective. In addition, a Zn–Fe alloy coating with 0.9% Fe content was obtained on the sintered NdFeB specimens. The coating thickness was about 10 μm. After electroplating, the coating was bright-dipped in the HNO_3_ solution with a volume fraction of 0.5% for 3–4 s at room temperature, rinsed in deionized water, and then passivated by dipping in a Cr(III) solution. Details of this process were published in our patent [44].

### 2.4. Testing

The surface morphology of the coatings was obtained by a scanning electron microscope (SEM) equipped with an energy dispersive spectrometer (EDS, S-4300, Hitachi) and a three-dimensional (3D) white-light interfering surface profiler (Micro XAM-3D). The element distribution in passivation film was characterized by the electron probe micro-analyzer (EPMA, JEOL JXA8530F). The phase structure of the coatings was analyzed by the X-ray diffractometer (XRD, D/max 2500) with Cu K_a_ radiation.

Electrochemical tests were performed using a classical three-electrode system with platinum as counter electrode, saturated calomel electrode SCE as a reference electrode, and a specimen with an exposed area of 1 cm^2^ as the working electrode. The test electrolyte was neutral 3.5 wt.% NaCl solutions, and the test temperature was maintained at 30 °C.

The electrochemical behavior was investigated by the cyclic voltammetry (CV), potentiodynamic polarization measurements and electrochemical impedance spectroscopy (EIS) utilizing an electrochemical workstation containing Potentiostat/Galvanostat (273 A, Princeton) and Frequency Response Detector (FRD100, Princeton). The basic electrolyte (BE) composition of CV test was shown in Table 2. Then, the ZnCl_2_ (60 g/L), FeSO_4_ (10 g/L) and ZnCl_2_ (60 g/L) + FeSO_4_ (10 g/L) were added into BE for a comparison study of the different electrodeposition processes, respectively. The sweep potential range of CV was derived from the open circuit potential up to −1.7 V with a sweep rate of 10 mV/s. At the same time, the experimental temperature and pH are compatible with the electrodeposition conditions, as shown in Table 1.

The constant voltage sweep rate for the potentiodynamic polarization measurements was set to 1 mV/s. The specimen was kept in a solution for 0.5 h prior to the start of the test, while the initial potential for the polarization curve was an open circuit potential. The potential was scanned in the cathodic direction and from the open circuit potential in the direction of increasing polarization. In addition, the test magnet matrix was not coated.

In addition, the impedance measurements were performed with a sinusoidal signal with an amplitude of 5 mV in the frequency range of 10^5^ to 10^−2^ Hz. The impedance data were then fitted and analyzed using the ZsimpWin software. Finally, the neutral salt spray (NSS) test was performed in a standard fog chamber with the 3.5 wt.% NaCl solutions at 30 °C to evaluate the corrosion resistance of various coatings.

## 3. Results and Discussion

### 3.1. SEM Analysis

**Figure 1:**
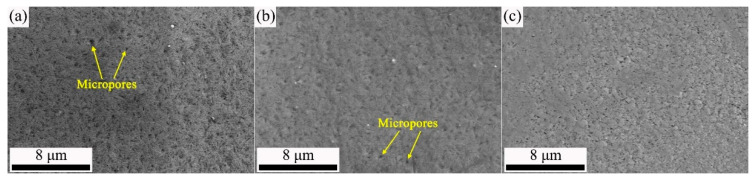

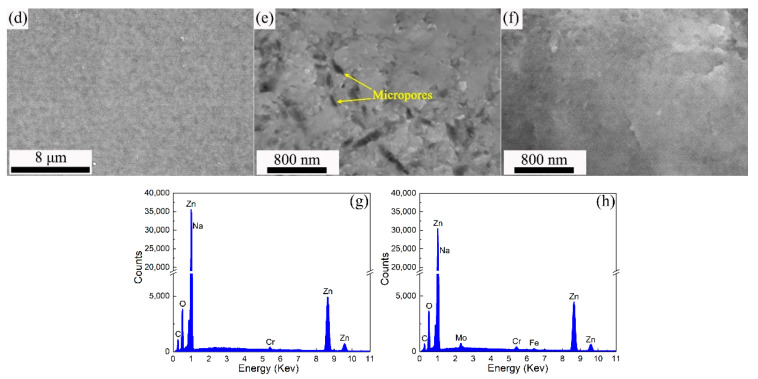
SEM images of: (**a**) ZC; (**b**) ZFC; (**c**) PZC and (**d**) PZFC, (**e**,**f**) are the magnified view of (**c**,**d**), (**g**) EDS of PZC, (**h**) EDS of PZFC.

The surface morphology of the Zn coating (ZC), Zn–Fe alloy coating (ZFC), passivated Zn coating (PZC) and passivated Zn–Fe alloy coating (PZFC) on NdFeB substrates are shown in Figure 1. When comparing ZC with ZFC specimens, as shown in Figure 1a,b, both of their coating surfaces had a number of micropores without microcracks. Most of the defects were caused by the sintering process of NdFeB, and a small fraction was due to the intrinsic defects in the coating. Then, the corrosion medium could easily and preferentially move towards these micropores, leading to the dissolution of the pore inwall, which could expand the pore and cause further concentration of the corrosion medium. Nevertheless, the micropore number of the ZFC was less than that of the ZC, indicating that the ZFC had a denser microstructure. A denser microstructure of the ZFC is beneficial for obtaining a more compact passivation film. The Cr(III) passivation film lacks a self-repairing capability. Therefore, in order to improve the corrosion resistance of the passivated film, the micropore number should be reduced as much as possible. Figure 1c,d shows the morphology of PZFC and PZC; meanwhile, the corresponding magnified images are presented in Figure 1e,f. It can be seen that the micropores in ZFC were effectively filled and overlaid by the Cr(III) passivation film which could inhibit the corrosion process of coating to a certain extent through holding back the transfer of corrosion medium. However, the PZC still had numerous micropores, although their number and size decreased. Furthermore, the Cr content in the PZC and PZFC was 1.0% and 1.1%, respectively, as shown in Figure 1g,h.

### 3.2. D White-Light Interfering Surface Analysis

**Figure 2:**
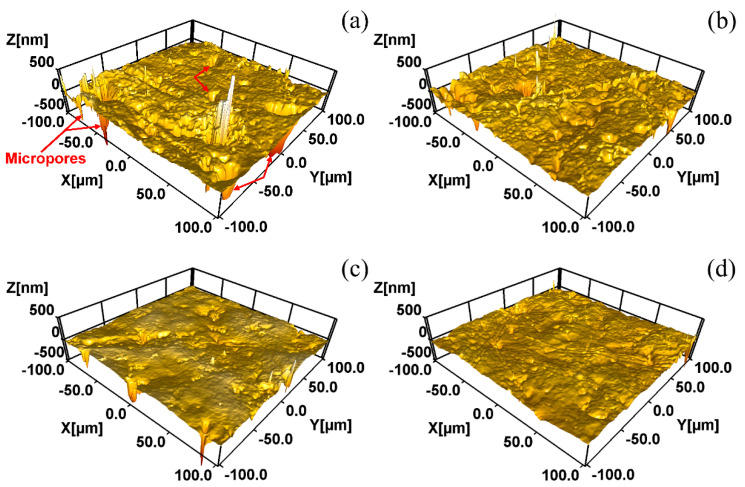
Coating surface morphologies of specimens from 3D white-light interfering surface profiler: (**a**) ZC; (**b**) ZFC; (**c**) PZC; (**d**) PZFC.

Figure 2a–d displays the high-resolution 3D image of the various coatings acquired by a 3D white-light interfering surface profiler. It can be clearly seen that the micropores (marked by red arrows) of ZC were larger and deeper than those of the ZFC. Then, the PZFC showed a more uniform and smooth surface morphology than the PZC. Moreover, the measured roughness values were 68.1 nm, 44.5 nm, 55.3 nm and 36.4 nm for ZC, ZFC, PZC and PZFC, respectively. The reduction in roughness after passivating indicated that the passivation process was able to fill in the surface defects of the coating to a certain extent. Compared with the SEM images, the 3D images present a stereoscopic structure of the coating surface, which directly characterizes the morphology of the various coatings.

### 3.3. XRD Analysis

**Figure 3:**
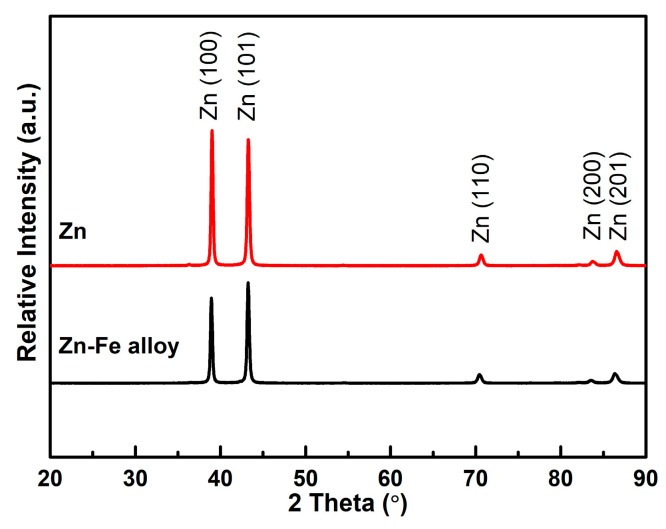
XRD spectra of ZC and ZFC.

As shown in Figure 3, the XRD patterns of the ZFC and ZC were almost similar, and both of them contained the Zn (100), (101), (110), (200) and (201) planes. A small difference was the peak intensity of the ZFC, which was slightly lower than the ZC. In this case, it demonstrates that the η-phase with hcp structure is the single phase in both ZC and ZFC [45]. The single-phase structure can avoid microporous corrosion to a certain extent compared with the multiphase structures that exist in Zn–Fe alloy coatings with a high iron content [46].

### 3.4. EPMA Analysis

**Figure 4:**
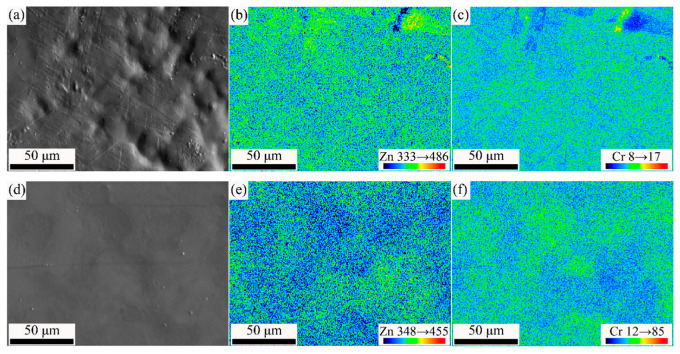

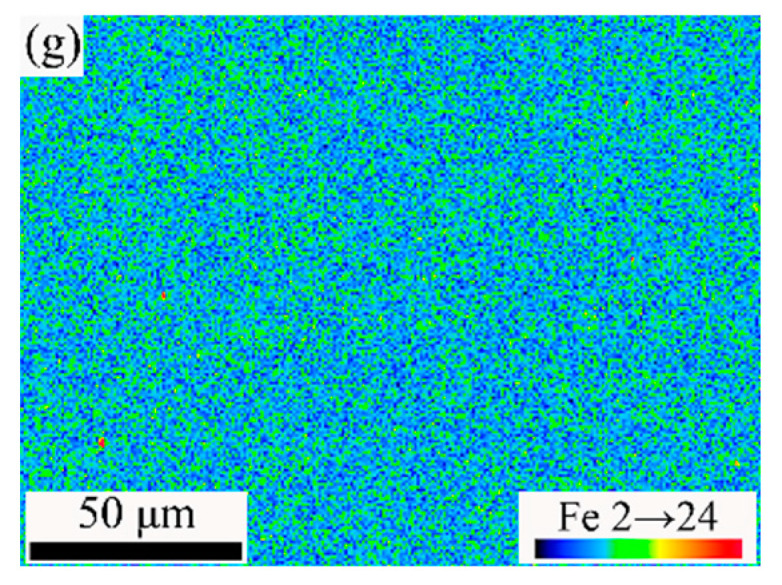
EPMA images with large size of passivated film: (**a**–**c**) SEM, Zn and Cr mapping of PZC, (**d**–**g**) SEM, Zn, Cr and Fe mapping of PZFC, respectively.

Figure 4 shows the EPMA images of PZC and PZFC. As displayed in Figure 4a,c of the SEM morphology, the PZC surface was uneven, while the PZFC had a very flat surface. The distribution of both Zn and Cr elements in the PZC was uniform as a whole. In contrast, the intensity of the Zn and Cr elements was complementary, as shown in Figure 4e,f. Therefore, the Cr elements were enriched in the concave position of the PZFC, showing a micro-leveling effect. Moreover, the Fe elements were uniformly distributed in the PZFC, as seen in Figure 4g.

### 3.5. Cyclic Voltammogram Analysis

**Figure 5:**
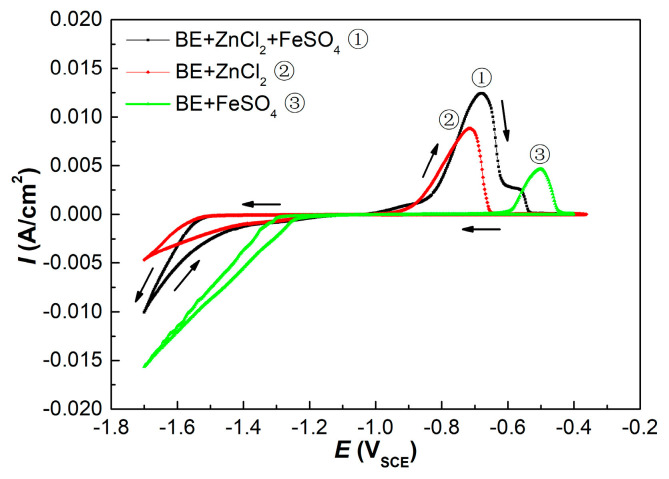
Cyclic voltammograms of different electrodeposition process (① BE + ZnCl_2_ (60 g/L) + FeSO_4_ (10 g/L); ② BE + ZnCl_2_ (60 g/L); ③ BE + FeSO_4_ (10 g/L). The working electrode of the CV test was a copper electrode).

In this study, the cyclic voltammogram indicated the electrodeposition processes of the Zn^2+^, Fe^2+^ and Zn^2+^ + Fe^2+^ ions, respectively. As shown in Figure 5, there was current loop in the cathodic branch of all the three voltammograms, which indicated the nucleation and grain growth process [28,47]. The electrodeposition initial potential of Zn–Fe alloy was similar to that of pure Zn, but the curve slope was larger than that of the latter, which demonstrated that the nucleation rate of ZFC was faster than ZC [48]. The addition of Fe in the ZFC was responsible for its nucleation rate increase, which resulted from the largest curve slope for pure Fe in the three cyclic voltammograms. Zhang et al. also indicated that the Fe^2+^ ions could promote the co-deposition of Zn–Fe alloy [48]. Then, considering that the micropores of ZFC were less than those of ZC, as shown in Figure 1 and Figure 2, it can be concluded that the higher nucleation rates could refine the grain sizes that enhanced the coating compactness.

Moreover, the peak potentials for Zn and Fe alone were −718 mV and −501 mV, respectively, in the anodic branches of the cyclic voltammogram. The dissolution peak potential of the ZFC, however, shifted by 40 mV towards the positive direction with respect to the ZC, resulting from the more anodic potential of Fe. In addition, two dissolution peaks were found in the anodic branch of the ZFC. Accordingly, the second dissolution peak of the ZFC at −560 mV should be the dissolution of pure Fe. It has been confirmed by XRD that there was only one phase in the ZFC. Consequently, it can be inferred that Fe in ZFC was predominantly in a solid solution in the η-phase and present in a small amount in elemental form, while due to the low Fe content (0.9 wt.%) in the alloy coating, the slight amount of Fe in elemental form was not identified by XRD.

### 3.6. Potentiodynamic Polarization Analysis

**Figure 6:**
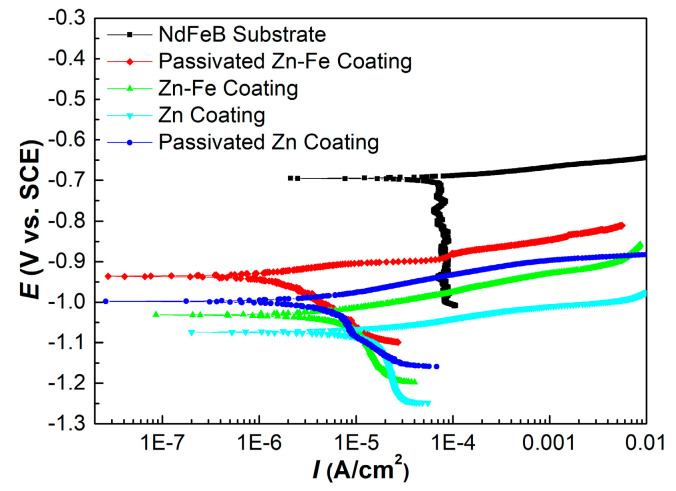
Polarization curves of different specimens in natural 3.5 wt.% NaCl solution.

Typical potentiodynamic polarization curves for different specimens were presented in Figure 6. It can be seen that all of the five curves exhibited active dissolution without any distinctive transition to passivation within the studied range of the anodic potential. There was no repassivation feature of coatings indicating that no Cr (VI) species were present in both PZC and PZFC. In addition, both the pure Zn system (ZC and PZC) and Zn–Fe alloy system (ZFC and PZFC) were anodic coatings relative to the NdFeB substrate.

Different electrochemical parameters, including corrosion potential and corrosion current density derived from the polarization curves, are summarized in Table 3. The corrosion potential of ZFC was nobler than that of ZC, and the corrosion current density of ZFC was approximately one order of magnitude lower than that of ZC, clearly demonstrating that the corrosion rate of ZFC was significantly reduced by the addition of iron. Meanwhile, the corrosion potential of PZFC was enhanced by 98 mV compared to ZFC, and the corrosion current density of PZFC was approximately one-fourth that of ZFC, indicating that the passivation was able to significantly improve the corrosion resistance of the coating. The variation rule of the pure Zn system resembled that of the Zn–Fe alloy system. In particular, the PZFC had the noblest corrosion potential and the lowest corrosion current density.

From the above analysis, it can be concluded that both the Zn–Fe alloy system and the pure Zn system exhibit fine protection for the substrate, but the nobler corrosion potential and much lower corrosion current density for the Zn–Fe alloy system indicate better protection for the NdFeB substrate. Furthermore, it also can be expected that the corrosion resistance of the Cr(III) film can be enhanced by reducing the micropore density of coating.

### 3.7. EIS Analysis

**Figure 7:**
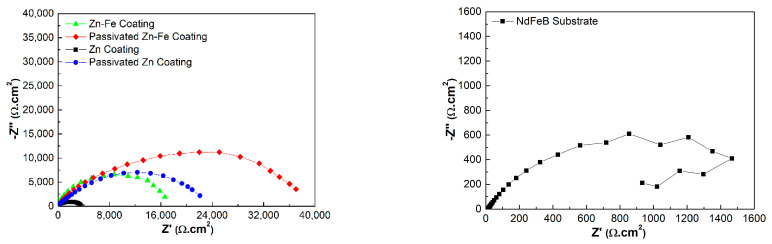
Nyquist impedance plots of different specimens in neutral 3.5 wt.% NaCl solution.

**Figure 8:**
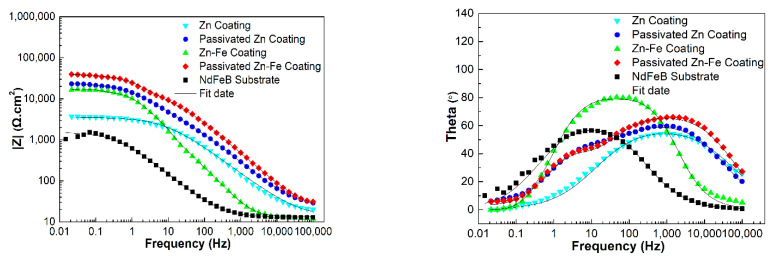
Bode impedance plots of different specimens in neutral 3.5 wt.% NaCl solution.

**Figure 9:**
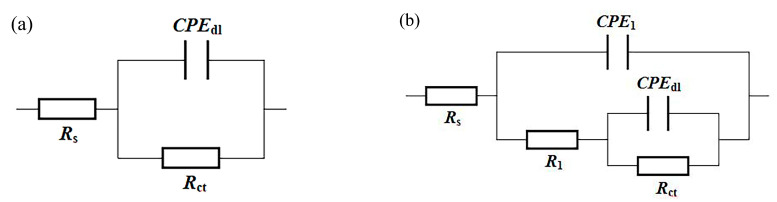
Electrochemical equivalent circuits used for fitting the EIS data: (**a**) NdFeB substrate, ZC and ZFC; (**b**) PZC and PZFC.

The comparisons of EIS dates for different specimens are displayed in Figure 7 and Figure 8. It is known that the capacitance loop diameter of the Nyquist impedance plot may reflect the corrosion resistance of the coating. The larger the capacitance loop diameter, the better the coating anticorrosive capacity [23]. Therefore, the corrosion resistance from high to low was PZFC, PZC, ZFC and ZC, respectively, as shown in Figure 7. This result was consistent with the conclusions of the analysis of the potentiodynamic polarization. Figure 8 shows the Bode plots of different specimens. It can be seen that there was only one peak over the whole frequency for the NdFeB substrate, ZC and FZC in the phase angle plot of Bode plot, which indicated one-time constants corresponding to one resistance–capacitance (RC) equivalent circuit. Consequently, a simple equivalent circuit, depicted in Figure 9a, was proposed to account for the EIS dates of the three circuits. In this equivalent circuit, *R_s_* represents electrolyte resistance, *R_c_*_t_ represents the charge transfer of the electrode reactions, and a constant phase element (*CPE_dl_*) was used to replace the capacitance of the double layer (C*_dl_*) due to the non-ideal capacitive response of the system. The impedance of a CPE is given by the following equation [49]:

(1) $$Z_{CPE}=Y_{0}^{-1}{\left(jw\right)}^{-n}$$

where *Y*_0_ is the admittance constant of the CPE, *ω* is the angular frequency, *j* = $\sqrt{-1}$ and *n* is the *CPE* exponent. The capacitance of the double layer is estimated using the following equation [50]:

(2) C=Y01nR(1−n)n

where *R* is the resistance parallel to the *CPE*.

Then, as shown in Figure 9b, the equivalent circuit consisting of two RC circuits was used to interpret the EIS dates of PZC and PZFC, resulting from two peaks in their phase angle plots. This indicates that passivation treatment alters the electrochemical behavior of the coating. In this case, *R_1_* and *CPE_1_* represents the resistance and capacitance response of the passivation layer, respectively. *R_ct_* modeled the charge transfer resistance, and *CPE_dl_* describes the double layer capacitance. As shown in Figure 8, the impedance dates were well-fitted using the two types of equivalent circuits.

The values of the electrochemical parameters fitted by the equivalent circuit for the various coatings are all listed in Table 4. It was found that the *R_ct_* of ZFC was approximately four times higher than that of ZC, which should be due to the dispersive distribution of iron in the coating suppressing the charge transfer process. Then, the total polarization resistance (*R_1_* + *R_ct_*) of PZC and PZFC dramatically increased, which implied that the charge transfer between the passivation layer and solution became more difficult. The double-layer capacitance is proportional to the area of the electrochemical reaction such as pore or microcrack in contact with the aggressive environment [51]. Therefore, the high *C_dl_* values of NdFeB could be associated with the local corrosion occurring at the electrolyte/metal interface due to its intrinsic defects. The *C_dl_* of ZC and ZFC decreased by approximately one order of magnitude compared with the NdFeB substrate, indicating that the inherent defects in the surface area had been efficiently filled by the leveling action of the brightener. Then, the *C_dl_* of PZC and PZFC was further decreased by several orders of magnitude compared with the values of ZC and ZFC, implying that the corrosion inside the coating defects of PZC and PZFC became less severe due to the reduction in the surface area of the micropore after the passivating process. Moreover, the *C_dl_* of ZFC was lower than that of the ZC due to the more compact coating of the ZFC caused by its high electrodeposition efficiency. It was reported that the Zn–Fe alloy coating should have low iron content (about 1 wt.% Fe) for obtaining a good receptivity to passivate [32]. The existence of iron in an elemental form is beneficial for its diffusion and enrichment at the defect position [50]. Therefore, the C*_dll_* of PZFC was one fifth that of PZC, and the total resistance (*R_1_* + *R_ct_*) of PZFC was about 1.7 times that of PZC, which was likely to be related to a surface iron enrichment. Thus, the defect surface area can be padded by this element enrichment process promoting the formation of the compact passivation film, which can act as a barrier layer to inhibit the anodic dissolution on the whole coating surface. Finally, the total anticorrosive capacity of the coating is extremely high. This has also been proved by the NSS tests that PZFC can withstand 210 h against a neutral 3.5 wt.% NaCl salt spray without any white rust, which was 3–4 times longer than that of PZC.

## 4. Conclusions

In summary, the following conclusions have been drawn from the above investigation.

(1) Both pure Zn and Zn–Fe alloy systems could provide efficient protection for the NdFeB substrate; nevertheless, the Zn–Fe alloy system displayed more excellent corrosion resistance. The Cr(III) passivated Zn–Fe alloy coating (PZFC) exhibited the highest corrosion potential, lowest corrosion current density and highest electrochemical impedance, which was attributed to the low-iron content (about 0.9 wt.%) in the coating.
(2) The Fe element in Zn–Fe alloy coating was predominantly in solid solution in η-phase and had small amounts in elemental form.
(3) The C*_dll_* of PZFC was one fifth that of PZC, and the total resistance (*R_1_* + *R_ct_*) of PZFC was about 1.7 times that of PZC, which could provide a physical barrier function for the sintered NdFeB substrate.
(4) The PZFC withstood 210 h against neutral 3.5 wt.% NaCl salt spray without any white rust, which was 3–4 times longer than that of the PZC.

## Figures and Tables

**Table 1:** Bath composition and electrodeposition parameters.

Component	Pure Zn	Zn–Fe
FeSO_4_·7H_2_O (g/L)	/	10
ZnCl_2_ (g/L)	60	60
KCl (g/L)	210	210
H_3_BO_3_ (g/L)	28	28
C_6_H_11_NaO_7_ (g/L)	/	8
C_4_O_6_H_4_KNa (g/L)	/	10
Ascorbic acid (g/L)	/	1
Additive (mL/L)	20	20
pH	4.5–5.5	4.5–5.5
Current density (A/dm^2^)	1.5	1.5
Temperature (°C)	30	30
Time (min)	45	45

**Table 2:** Basic electrolyte for cyclic voltammetry test.

Component	g/L
KCl	210
H_3_BO_3_	28
C_6_H_11_NaO_7_	8
C_4_O_6_H_4_KNa	10
Ascorbic acid	1
Additive	20 mL/L

**Table 3:** Electrochemical parameters calculated from polarization curves.

Specimens	Corrosion Potential (mV vs. SCE)	Corrosion Current Density (A cm^−2^)
PZFC	−936	0.7 × 10^−6^
PZC	−998	3.3 × 10^−6^
ZFC	−1032	4.1 × 10^−6^
ZC	−1074	1.9 × 10^−5^
NdFeB Substrate	−695	6.6 × 10^−5^

**Table 4:** Electrochemical parameters fitted by equivalent circuit.

Specimens	*R_1_* (Ω cm^2^)	*Y_o1_* (Ω^−1^ cm^−2^ s^−n^)	*n*	*R_ct_* (Ω cm^2^)	*Y_odl_* (Ω^−1^ cm^−2^ s^−n^)	*n*	*C_dl_* (F cm^−2^)
PZFC	8795	2.7 × 10^−6^	0.8	3.2 × 10^4^	2.1 × 10^−6^	0.8	1.1 × 10^−6^
PZC	5882	6.4 × 10^−6^	0.8	1.8 × 10^4^	8.4 × 10^−6^	0.8	5.2 × 10^−6^
ZFC				1.5 × 10^4^	1.3 × 10^−5^	0.8	8.7 × 10^−6^
ZC				3595	2.1 × 10^−5^	0.8	1.1 × 10^−5^
NdFeB Substrate				1775	3.5 × 10^−4^	0.7	2.9 × 10^−4^

## Data Availability

We confirm that the data supporting the findings of this study are available within the article.
